# Role of the hydrolytic-acidogenic phase on the removal of bisphenol A and sildenafil during anaerobic treatment

**DOI:** 10.1007/s10661-023-12009-8

**Published:** 2023-11-30

**Authors:** Jennifer Arcila-Saenz, Gina Hincapié-Mejía, Yudy Andrea Londoño-Cañas, Gustavo A. Peñuela

**Affiliations:** 1 GDCON Research Group, Faculty of Engineering, University Research Headquarters (SIU), University of Antioquia, Street 70 #, 52-21 Medellín, Colombia; 2 Environment, Habitat and Sustainability Research Group, University Institution Colegio Mayor de Antioquia, Street 78 # 65 –, 46 Medellín, Colombia

**Keywords:** Anaerobic digestion, Biodegradation mechanisms, Biological treatments, Hydrolytic processes, Micropollutants, Wastewater treatment

## Abstract

This paper presents the main results of the removal of two pharmaceutical and personal care products (PPCPs), bisphenol A (BPA) and sildenafil (SDF), by applying anaerobic biological batch tests. The biomass used was previously acclimatized and the experiment lasted 28 days. The effect of factors such as compound (BPA and SDF), concentration and type of inoculum was assessed, considering the factorial experimental design. The results indicated that evaluated factors did not significantly affect the PPCPs elimination in the evaluated range with a confidence level of 95%. On the other hand, the removal percentages obtained with BPA were mainly related to mechanisms, such as sorption and abiotic reactions. Regarding SDF, biodegradation was the predominant mechanism of removal under the experimental conditions of this study; however, the degradation of SDF was partial, with percentages lower than 43% in the tests with hydrolytic/acidogenic inoculum (H/A) and lower than 41% in the tests with methanogenic inoculum (MET). Finally, these findings indicated that hydrolysis/acidogenesis phase is a main contributor to SDF biodegradation in anaerobic digestion. The study provides a starting point for future research that seeks to improve treatment systems to optimize the removal of pollutants from different water sources.

**Supplementary Information:**

The online version contains supplementary material available at 10.1007/s10661-023-12009-8.

## Introduction

Water is a vital component in earth and is an ecological and economic resource of great importance, which as a result of rapid population growth and inadequate use has suffered an alarming deterioration (Barceló & López, 2010; Magro et al., 2020). In this sense, the greatest source of pollution in aquatic systems derives from the emissions of wastewater, which contain many substances of various kinds. One of the main causes of water quality deterioration are chemical pollutants used in domestic, agricultural, and industrial activities (Vasilachi et al., 2021). In the last two decades, there has been a growing concern about the detection of organic pollutants or “emerging pollutants” in environmental matrices, and studies in this area have increased (Verlicchi et al., 2010). Although their presence in the environment is not new, there are concerns regarding their possible consequences for health and the different ecosystems (Shahid et al., 2021; Vasilachi et al., 2021).

Emerging pollutants or micropollutants include a variety of pharmaceuticals and personal care products known as PPCPs; these include analgesics, anti-inflammatories, drugs for human and veterinary use, and cleaning, disinfection, and body care products (Huang et al., 2012; Yang et al., 2017). These pollutants are natural or synthetic compounds whose negative effects on humans and ecosystems are have just begun to be established (Rodriguez-Narvaez et al., 2017). Although a large proportion of emerging pollutants are found in low concentrations (ng to μg/L), some of them have the potential to accumulate and reach concentrations that could have potentially toxic effects on aquatic ecology and human health (Delgadillo-Mirquez et al., 2011; Geissen et al., 2015; Toušová et al., 2019). Methodologies for their quantification have been reported, but despite this, information regarding their presence and impacts in different environmental compartments is limited, which is used to justify the fact that they have not been regulated (Geissen et al., 2015; La Farré et al., 2008; Luo et al., 2023).

Bisphenol A (BPA) is a synthetic organic compound that belongs to the category of xenohormones and has also been classified as PPCP (Dodgen et al., 2014; Hernandez-Ruiz et al., 2012; Porter et al., 2020; Qin et al., 2015). These compounds act as endocrine disruptors and could modify hormone production or activity. In recent years, a certain relationship between exposure to endocrine disruptors and the generation of changes in metabolism, development, growth, and reproduction of organisms has been established (Cartagena, 2011). BPA is mainly used as an intermediary in the production of polycarbonate plastics and epoxy resins, and is also widely used for the manufacture of different products used in daily life (Huang et al., 2012). Although its toxicity has been studied and it has been shown that it can be metabolized by some aquatic organisms, research is required into the degradation of BPA by a wide range of higher organisms, as well as into the efficiency of treatments for their water removal (Kang et al., 2008).

Sildenafil (SDF) is a drug classified as a selective phosphodiesterase inhibitor type 5 (PDE5). It is used as a cardiovascular agent, causing smooth muscle relaxation, in particular in the pulmonary vasculature and the corpus cavernosum (Croom & Curran, 2012). PDEs are essential isoenzymes, and the regulation of these is integral to controlling physiological functions (Seftel, 2004). This drug causes pulmonary vasodilation in patients with pulmonary hypertension (Croom & Curran, 2012). SDF is also indicated as a treatment for erectile dysfunction; this compound allows the smooth muscle of the cavernous body to relax, enhancing erections during sexual stimulation (Felice et al., 2009; Salonia et al., 2003). SDF is within the group known as “lifestyle medications,” sildenafil is sold without medical prescription, and its consumption has increased to a level that it has been detected in water resources and effluents from wastewater treatment plants and even water for human consumption (Delgado et al., 2020). Additionally, there is still little information reported on the potential effects of SDF on the environment and wildlife (Herbert et al., 2015). Therefore, it is necessary to carry out investigations on the impacts of this compound and to develop specific treatment systems that allow its removal from water to be optimized.

Multiple studies on micropollutants indicate that they were not removed or were partially removed in conventional treatment systems (Gogoi et al., 2018). The compounds tested have different physicochemical characteristics, which means that the elimination mechanisms depend on factors such as the nature of the compound, octanol/water coefficient, sorption capacity, and concentration. For these reasons, their removal behavior differs markedly in biological systems. Moreover, the effects that each pollutant can have on microbial populations present in reactors vary (Rodríguez, Londoño & Peñuela, 2017; Wang & Wang, 2016).

Microbial degradation is the most important mechanism for removing pollutants in the environment since microorganisms have the potential to use these compounds as co-substrate depending on their metabolic development. Although strictly anaerobic conditions prevail in several environmental compartments, so far biodegradation studies on emerging organic pollutants such as PPCPs have focused mainly on aerobic conditions. However, certain recalcitrant pollutants biodegrade under strictly anaerobic conditions and little is known about the organisms and enzymatic processes involved in their biodegradation (Ghattas et al., 2017; Londoño, 2018).

Previous studies have reported high removal efficiencies of PPCPs in anaerobic systems, demonstrating the potential of such processes in the degradation of pollutants (Alvarino et al., 2014; Arias et al., 2018; Phan et al., 2018). It has been reported that the presence of certain consortiums of anaerobic microorganisms can enhance the biodegradation of some PPCPs, probably because the presence of biodegradable carbon sources stimulates the synthesis of enzymes that are also involved in the transformation of some micropollutants (Carneiro et al., 2020). In this sense, enzyme induction of microorganisms seems to be a key aspect of biodegradation, which has been insufficiently investigated (Gonzalez-Gil et al., 2019).

Therefore, the objective of this study is to ascertain the contribution of the hydrolytic/acidogenic phase and the overall anaerobic digestion process to the removal of sildenafil (SDF) and bisphenol A (BPA). With this aim, the effect of different factors on the biodegradation of PPCPs was evaluated. The experiment was carried out by applying batch tests with previously acclimatized biomass in expanded granular sludge bed reactors (EGSB).

## Materials and methods

### Chemical standards

SDF and BPA chemical standards with a purity > 95% were purchased from Merck. Total organic carbon (TOC) standard solution with a purity > 95% was purchased from Sigma-Aldrich. Volatile fatty acids mix chemical standard with a purity > 95% was purchased from Restek Corporation. For the ultra-high performance liquid chromatograph (UHPLC) assays, acetonitrile, acetic acid, and methanol LC-MS grade and of 99.9% purity were purchased from Merck.

### Batch experiments

Previously acclimatized anaerobic biomass from an EGSB methanogenic reactor was used for degradation tests with all consortiums of microorganisms involved in anaerobic digestion (hydrolytic, acidogenic, acetogenic, and methanogenic microorganisms). These consortiums were abbreviated as methanogenic biomass (MET). Additionally, previously acclimatized biomass from an EGSB reactor operated under hydrolytic conditions was used for degradation tests with hydrolytic/acidogenic consortiums (H/A). EGSB anaerobic reactors were installed in the laboratory of the Diagnostic and Pollution Control Research Group (GDCON) of the University of Antioquia, Colombia. Tests performed are summarized in Table 1.

**Table 1:** Experimental design for batch tests with hydrolytic/acidogenic and methanogenic biomass

Test	Hydrolytic/acidogenic or methanogenic test description
Blank/control	Inoculum + culture medium without substrate
Control	Inoculum + culture medium
Blank B1_50_BPA	Sterile culture medium + C50 μg/L
Blank B2_500_BPA	Sterile culture medium + C500 μg/L
Blank B3_50_SDF	Sterile culture medium + C50 μg/L
Blank B4_500_SDF	Sterile culture medium + C500 μg/L
Blank B5_50_BPA	Sterile inoculum + sterile culture medium + C50 μg/L
Blank B6_500_BPA	Sterile inoculum + sterile culture medium + C500 μg/L
Blank B7_50_SDF	Sterile inoculum + sterile culture medium + C50 μg/L
Blank B8_500_SDF	Sterile inoculum + sterile culture medium + C500 μg/L
Sample M1_50_BPA	Inoculum + culture medium + C50 μg/L
Sample M2_500_BPA	Inoculum + culture medium + C500 μg/L
Sample M3_50_SDF	Inoculum + culture medium + C50 μg/L
Sample M4_500_SDF	Inoculum + culture medium + C500 μg/L

Abbreviations: *C50*, concentration of 50 μg/L; *C500*, concentration of 500 μg/L; *Blank/Sample #_50/500_BPA/SDF*, type of test/test number_concentration compound_PPCP (BPA or SDF)

The assays were performed using 60-mL serological bottles. Two culture mediums were prepared to ensure adequate environmental conditions for both methanogenic and hydrolytic microorganisms. A culture medium was prepared with 50 mL of macronutrients, 10 mL of micronutrients, 0.3 g of yeast extract, and 1 mL of 0.1% resazurin per liter of culture medium. Subsequently, 0.5 g of cysteine and 2 g of NaHCO_3_ were added per liter of medium (Rodríguez et al., 2017). The pH was adjusted in a range of 6.8–7.2 and 4.5–5.5, for experiments with methanogenic and hydrolytic/acidogenic inoculum, respectively.

To each of the bottles, 43.2 mL of culture medium, 3 mL of anaerobic sludge (MET or H/A inoculum), and 0.3 mL of stock solution of each PPCP were added. Then, the bottles were sealed, and 1.5 mL of Na_2_S·9H_2_O (4 g/L) was added. The bottles were purged with nitrogen and left overnight for incubation at 37 °C under continuous agitation at 100 rpm. Next, 12 mL of the substrate solution (10.000 mg/L) was added; the substrate in this experiment was potato starch. Finally, the bottles were incubated again under the same conditions for 28 days. Starch was selected as a substrate since it is a readily biodegradable carbon source. Additionally, the project that funded this research was focused on studying the removal of organic matter from wastewater of the starch industry.

On days 0 and 28 of the experiment, sampling was carried out in each experimental unit (MET and H/A) to collect the results. The variables pH; total organic carbon (TOC); volatile fatty acids (VFA)—acetic, propionic, butyric, isobutyric, valeric, isovaleric; and concentrations of bisphenol A and sildenafil were measured. The pH was monitored and controlled continuously to keep the culture medium within the proposed pH range.

### Inoculum

The inoculum for EGSB systems was obtained from the upflow anaerobic sludge blanket (UASB) reactor of a wastewater treatment plant of the Colanta dairy company, located in the municipality of San Pedro, Antioquia (Colombia).

Total solids (TS) were determined in accordance with paragraph 2540 B “Total Solids Dried at 103-105°C” and volatile solids (VS) according to section 2540 E “Fixed and volatile Solids Ignited at 550°C” of the Standard Methods for the examination of water and wastewater (APHA, 2017). As the relative content of dissolved solids in the sludge used in the experiment was low with respect to suspended solids, volatile solids (VS) were used as a measure of the biomass present in the anaerobic sludge. Similarly, total solids (TS) were used as a measure of total suspended solids (TSS). The physicochemical characteristics of the inoculum were 48.32 g total solids (TS)/L; 36.26 g volatile solids (VS)/L; VS/TS ratio of 0.75; sludge volume index (SVI) of 23.60 mL/g; and a density of 23.91 g/L.

Subsequently, the biomass was acclimatized using methanogenic EGSB system; the reactor was operated with a pH range of around 7. This generated a balance in the reactor, which allowed the growth of microbial consortiums involved in anaerobic digestion, specifically, hydrolytic, acidogenic, acetogenic, and methanogenic microorganisms. On the other hand, the hydrolytic/acidogenic EGSB system was operated with a pH range around 5.0–5.5, which has been reported as the optimal range of hydrolytic and acidogenic microorganisms (Zhou et al., 2018); these conditions allowed the inhibition of methanogenic consortiums.

### Experimental design

The effects of three factors were assessed using a 2^3^-factorial design. Factor A (compound) had two levels: BPA and SDF, factor B (concentration of PPCPs) had two levels: 50 and 500 μg/L, and factor C (type of inoculum) had two levels: hydrolytic/acidogenic consortiums and the complete consortium of anaerobic digestion. The objective was to evaluate the influence of the factors on the removal of BPA and SDF.

The data were analyzed using Statgraphics Centurion XVI version 16.1.03 software (VA, USA), with a confidence level of 95%. The mean, standard deviation, and coefficient of variance were calculated. Multiple range tests were performed to find statistically significant differences between the means obtained for factors evaluated. Blank tests were performed for each of the studied pollutants to evaluate the presence of other non-biological removal mechanisms that may occur in the tests. The experiments were performed in triplicate. Tests performed are summarized in Table 1.

### Analytical methods

The identification and quantification of BPA and SDF in an aqueous solution was performed using an ACQUITY UPLC H-Class device (Waters, USA) coupled to a XEVO TQD mass spectrometer (Manchester, UK), UHPLC-MS/MS. The column used for chromatographic separation was a POROSHELL 120 (2.7 μm particle size, 100 Armstrong pore size, 100 × 2.1 mm, Agilent Technologies, USA). All data were acquired and processed using Masslynx v 4.1 software (Waters, USA). Mobile phase A consisted of water:acetic acid 0.1%, and mobile phase B consisted of acetonitrile:methanol (1:1). The mobile phase flow was set at 0.3 mL/min. The capillary voltage of Waters Xevo TQD was set to 2.25 kV. The source temperature was set at 150 °C and the desolvation gas temperature at 450 °C with a flow of 400 L/h and a cone gas flow of 50 L/h. The conditions mentioned were maintained for ESI (−) and ESI (+). Analysis run time was 11 min.

The quantification of volatile fatty acids (VFA) was performed by gas chromatography using a 6890 plus gas chromatograph (GC) (Agilent Technologies, USA) with a split/splitless injector 7683B (Agilent technologies, USA) and flame ionization detector (FID) (Agilent Technologies, USA). The column used for chromatographic separation was a DB-WAX device (1 μm, 30 m × 530 μm, Agilent Technologies, USA), and a constant flow of 5 mL/min helium gas at 99.999% purity was used. The oven was programmed to start at 80 °C for 1 min, ramp to 150 °C at 20 °C/min, and ramp to 190 °C at 5 °C/min. The detector temperature was set at 240 °C. The nitrogen flow was set at 20 mL/min. The hydrogen flow was set at 30 mL/min and the flow of air was set at 300 mL/min. Analysis run time was 12.50 min.

Analyses of total solids (TS), volatile solids (VS), sludge volumetric index (SVI), and pH were performed according to the protocols established in the Standard Methods (APHA, 2017). Total organic carbon (TOC) was analyzed according to the protocol established in ASTM D7573. The methodologies were performed in the GDCON group laboratory. The group is accredited by the Institute of Hydrology, Meteorology and Environmental Studies (IDEAM) to perform such analyses.

## Results and discussion

### Inoculum characterization for batch tests

Sludge used for both tests (MET and H/A) had adequate acclimatization time in the EGSB system. This allowed biomass to be obtained with high biological activity that favored the good performance of anaerobic systems. The ratios of VS/TS were 0.87 and 0.74 for H/A and MET biomass, respectively, indicating that approximately 87% and 74% of the total solid was organic in nature. The ratio allows an estimate of the high biological component of the inoculated sample in the experimental units.

### Volatile fatty acids and pH analysis

Figure S1 and Figure S2 (see Supplementary Information) show that the pH of the experimental units was within the proposed range for the H/A and MET experiments. However, it was necessary to dose a buffering agent for the controls and samples during the test because the pH of the medium decreased due to the production of VFAs. This explains the concentrations obtained of volatile fatty acids in the medium at the end of the experiment with H/A and MET inoculum (Figure S3 and Figure S4).

Hydrolysis and acidogenesis are the first stages of the anaerobic digestion of complex organic compounds. VFAs are the main products of organic matter acidogenesis, and their appearance indicates the presence and activity of hydrolytic and acidogenic microorganisms during the tests carried out (Parawira et al., 2004). For the H/A biomass experiment, VFAs produced were acetic, butyric, and propionic acid in a greater proportion (descending order), and i-butyric, valeric, and i-valeric acid in negligible quantities (Figure S3). It was observed that the concentration of VFAs in controls (without pollutants) and samples with BPA and SDF were similar, which suggests that none of the evaluated compounds affected hydrolytic/acidogenic consortiums. Additionally, acidogenic microorganisms were not inhibited with evaluated concentrations of pollutants. On the other hand, VFAs produced in the experiments with MET inoculum were butyric, acetic, and propionic acid in greater proportion (descending order), and valeric, i-valeric, and i-butyric acid in negligible quantities (Figure S4). Quantified VFAs concentrations at the end of the experiment suggest that the initial dissolved organic carbon (COD) was too high for the methanogenic inoculum supplied, considering that the experimental units were batch closed systems. In this way, the accumulation of VFAs probably triggered an imbalance in consortiums at some point in the experiment since methanogenic microorganisms did not transform all intermediate compounds into methane.

Many types of bacteria are involved in hydrolytic and acidogenic phases; therefore, different types of organic acids are produced according to factors such as substrate, physicochemical conditions of the medium, organic load, and hydraulic retention time (Zhou et al., 2018). According to a study by Parawira et al. (2004), acetic, butyric, and propionic acids are intermediate compounds produced mainly during the anaerobic fermentation of carbohydrates such as starch (the main source of carbon used in the experiments).

Additionally, it has been reported that pH can affect the type of VFAs produced during acidogenic fermentation (Lee et al., 2014). This behavior was also observed in experiments performed in this study because higher concentrations of acetic acid were produced in experimental units treated with inoculum H/A (medium with acid pH), while in experimental units with inoculum MET (medium with pH close to 7) higher concentrations of butyric acid were produced at the end of the experiment. The above could be explained by the structure of acetic acid (Hac), which can be converted to methane by methanogenic microorganisms (Parawira et al., 2004). This suggests that the low concentration of Hac at the end of experiments with methanogenic inoculum is probably related to its transformation into methane and, consistently with this, high concentration of Hac at the end of experiments with acidogenic inoculum is an indicator of the suppression of the methanogenic phase.

### Batch test

#### Biodegradability test for PPCPs

**Fig. 1:**
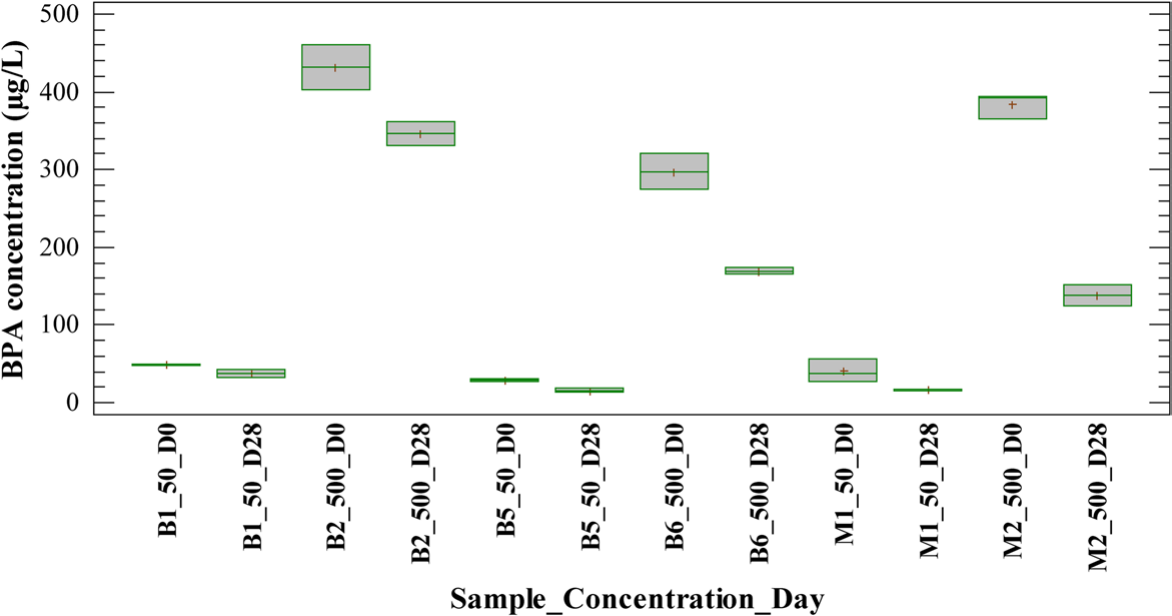
Behavior of the BPA concentration for blanks and samples with H/A inoculum in the anaerobic batch test

**Fig. 2:**
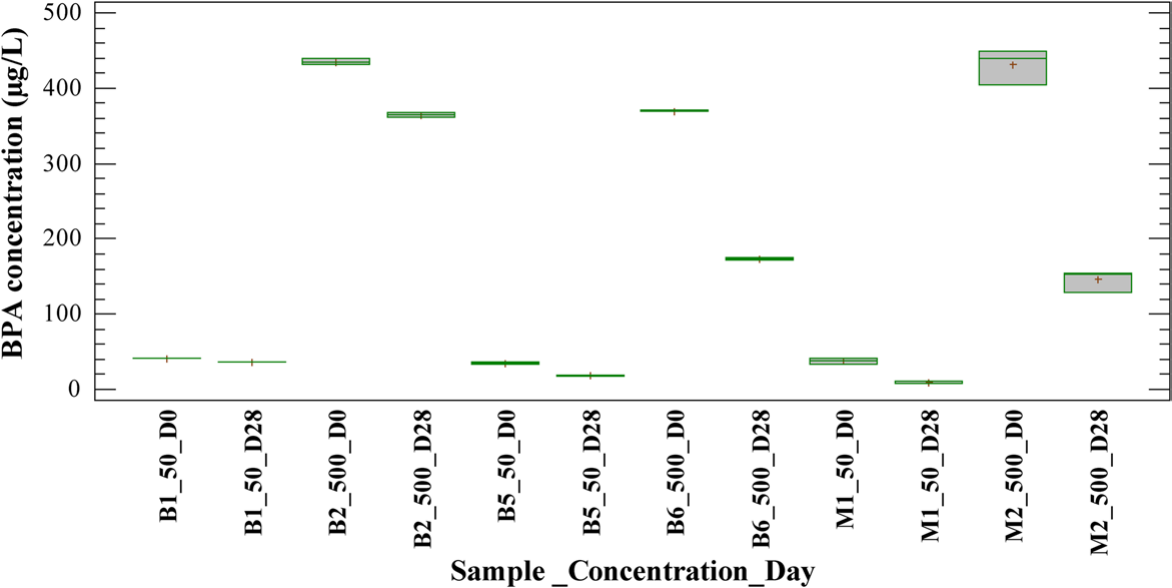
Behavior of the BPA concentration for blanks and samples with MET inoculum in the anaerobic batch test

Figures 1 and 2 show BPA concentration on days 0 and 28 of the experiment for blank and sample tests treated with H/A and MET inoculum, respectively. Blank tests were performed to verify whether the removal was related to biological mechanisms or alternative removal mechanisms such as sorption and hydrolysis. It is observed that BPA concentration decreased in blank tests 1 and 2 (B1_50_D28 and B2_500_D28) at the end of the experiment for concentrations evaluated. Considering that blank tests were performed only with sterile medium and PPCPs, this result was associated with a partial hydrolysis mechanism of BPA. This behavior was observed in tests with both types of inoculums, H/A and MET. The results were consistent with other reported studies on the behavior of BPA in environmental matrices. According to Cousins et al. (2010), bisphenol A degrades relatively quickly in the environment, with an overall half-life of 4.5 to 4.7 days depending on the release medium. BPA is considered a pseudo-persistent pollutant; nevertheless, its mass use as an intermediate in the production of polycarbonate and epoxy resins, flame retardants, and other mass use products has generated accumulation to the extent that it has been quantified at alarming levels in various environmental matrices (Gogoi et al., 2018; Maturi et al., 2023; Mohapatra et al., 2011).

Moreover, Figs. 1 and 2 show that BPA concentration decreased in blank tests 5 and 6 (B5_50_D28 and B6_500_D28) at the end of the experiment for both concentrations evaluated. Blank tests were performed using sterilized biomass, this suggesting that there were other removal mechanisms than biodegradation. The presence of other non-biological mechanisms was confirmed with the calculated removal percentages in the blank and sample tests. For the anaerobic test with H/A inoculum, the removal in blank tests 5 and 6 was between 43 and 43.5% with sterilized biomass, while in sample tests 1 and 2 it was between 59 and 63% with active biomass. Similarly, for the anaerobic test with MET inoculum, the removal in blank tests 5 and 6 was between 45 and 53% with sterilized biomass, while in sample tests 1 and 2 it was between 74 and 66% with MET active biomass. This result shows that the loss of analyte from the liquid phase at the end of the trials was mainly due to the sorption of BPA on the biomass. Therefore, sludge sorption was a predominant mechanism under the experimental conditions employed in both inoculums, H/A and MET.

Appreciable sorption/accumulation has been reported for log Kow values > 3 (Londoño & Peñuela, 2017). According to Cousins et al. (2010), the octanol-water coefficient of BPA (log Kow 4.3) causes the compound to have a lipophilic tendency, that is, it tends to be attached to organic phases such as soils and sediments, although a fraction of the compound may be present in the aqueous phase. Additionally, it has been reported that the BPA partition coefficient changes according to the redox conditions of the medium; thus, the value of the coefficient increases under anaerobic conditions. This explains the tendency of the compound to sorption during anaerobic tests performed (Wang et al., 2014). In summary, sorption on sludge is a mechanism that has been previously reported for the removal of multiple micropollutants in biological processes (Carneiro et al., 2020; Rodríguez et al., 2017).

According to what is discussed above, the results obtained with BPA for H/A and MET biomass treatments suggest that the removal percentages calculated in the experimental units with active biomass are mainly related to phenomena such as sorption on the biomass and abiotic reactions (in a lower proportion). This fact could also explain the low incidence of concentration in the removal of BPA. Similarly, it has been reported that BPA does not degrade under anaerobic conditions (Kang & Kondo, 2002; Limam et al., 2013).

**Fig. 3:**
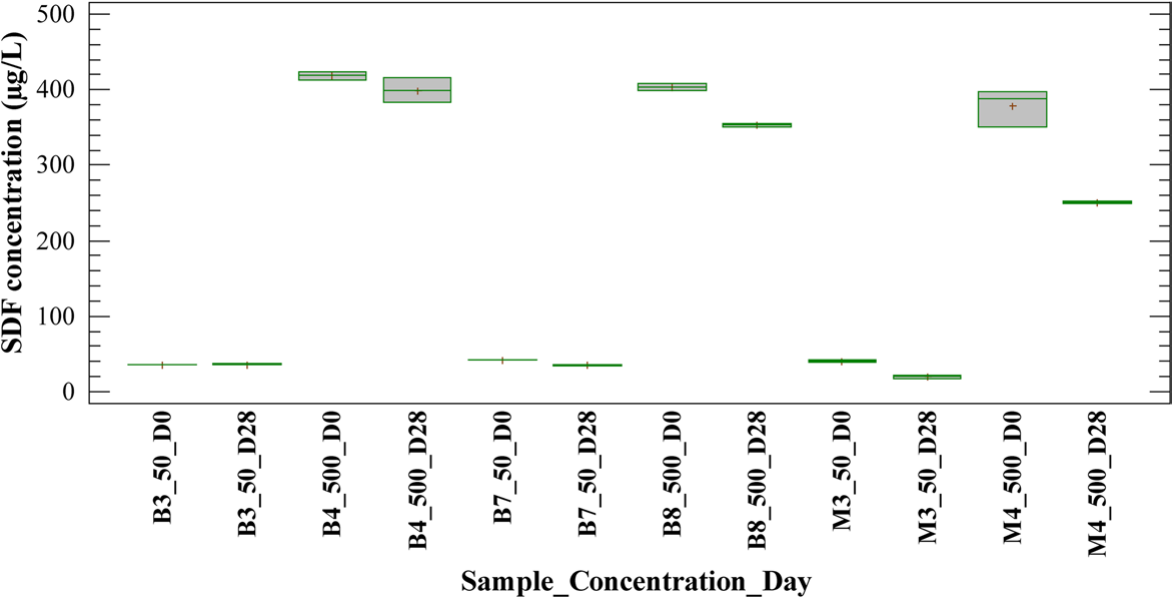
Behavior of the SDF concentration for blanks and samples with H/A inoculum in the anaerobic batch test

**Fig. 4:**
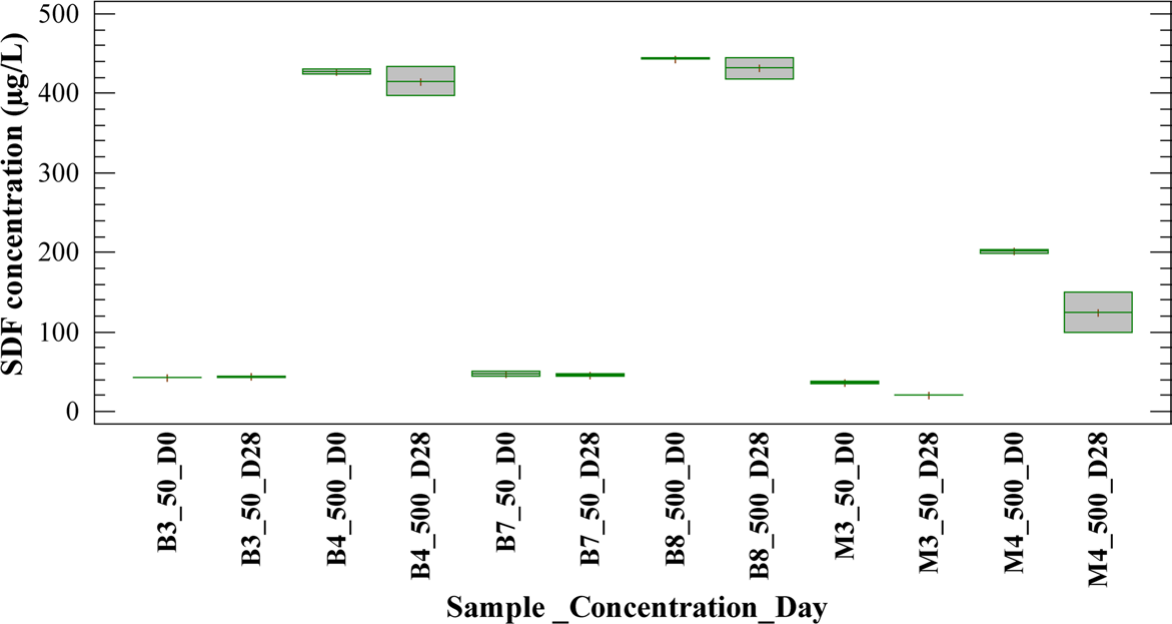
Behavior of the SDF concentration for blanks and samples with MET inoculum in the anaerobic batch test

Figures 3 and 4 show SDF concentration on days 0 and 28 of the experiment for blank and sample tests treated with H/A and MET inoculums, respectively. It is observed that, after 28 days, SDF concentration did not decrease in blank tests 3 and 4 (B3_50_D28 and B4_500_D28) for concentrations evaluated. This indicates that no hydrolysis mechanisms were present during the experiment with both types of inoculums, H/A and MET. On the other hand, blank tests 7 and 8 (B7_50_D28 and B8_500_D28) showed minimal changes in concentration at the end of the experiment. Therefore, sorption on the sludge was not an important mechanism of SDF under the experimental conditions of this study. These results were consistent with the partition coefficient reported for SDF (log Kow 1.9); the coefficient value indicates that this compound has a very low tendency to sorption on the biomass (Son et al., 2022).

According to what is discussed above, the removal obtained for sildenafil may have been related to a biodegradation mechanism. However, the degradation of the SDF was partial, with percentages lower than 43% in the tests with H/A inoculum and lower than 41% in the tests with MET inoculum. Considering that the removal obtained were similar with both types of biomass, the results suggested that hydrolysis and acidogenesis are a main contributor to SDF biodegradation. It could be expected that hydrolysis would be the most efficient phase to biodegradation SDF, so a huge number of extracellular enzymes are produced during starch fermentation and might have resulted in a greater cometabolic biotransformation of compound (Gonzalez-Gil et al., 2018). Nevertheless, Carneiro et al. (2020) indicated that hydrolysis of carbohydrates plays a minor role in the anaerobic biotransformation of some micropollutants, while acidogenesis should be considered as a key step. Overall, the scope of this study allows only concluding that hydrolysis/acidogenesis are involved in the anaerobic biotransformation of the SDF, while acetogenesis/methanogenesis have a low incidence on the biodegradation of this compound, under the experimental conditions of the experiments.

Removal efficiencies for SDF higher than 97% in planted wetlands and around 40% in artificial wetlands have been reported (Delgado et al., 2020). Though, simultaneous mechanisms may occur in wetlands, such as volatilization, sorption, sedimentation, photodegradation, plant uptake, and microbial degradation, which contribute to the removal or transformation of SDF. Additionally, the removal of sildenafil in wastewater treated with white rot fungi has been evaluated and biodegradation higher than 95% has been reported in previous studies (Tormo-Budowski et al., 2021). In contrast with the above, removal efficiencies for SDF of less than 20% have been reported in conventional treatment plants and biological fixed film treatments (Delgado et al., 2018, 2019). Also, Suanon et al. (2017) reported negative removal efficiencies for sildenafil in anaerobic digesters. In this context, it should be emphasized that there is little reported information about biological SDF degradation in aquatic ecosystems or treatment systems under anaerobic conditions.

### Effect of factors evaluated on PPCPs removal

**Fig. 5:**
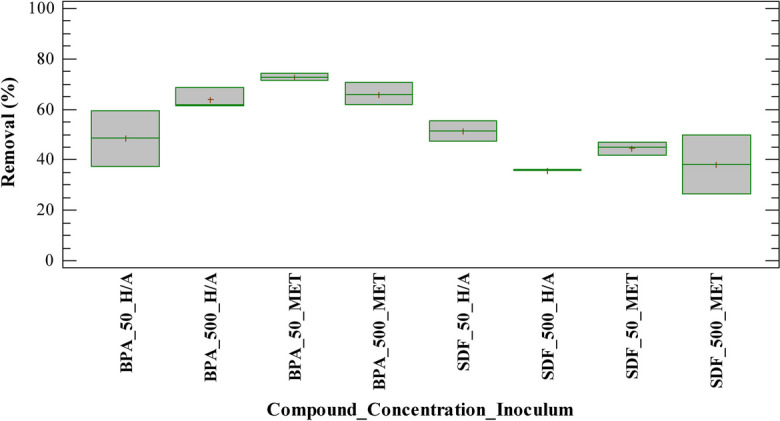
Removal of BPA and SDF with H/A and MET inoculum in the anaerobic batch test after 28 days

**Fig. 6:**
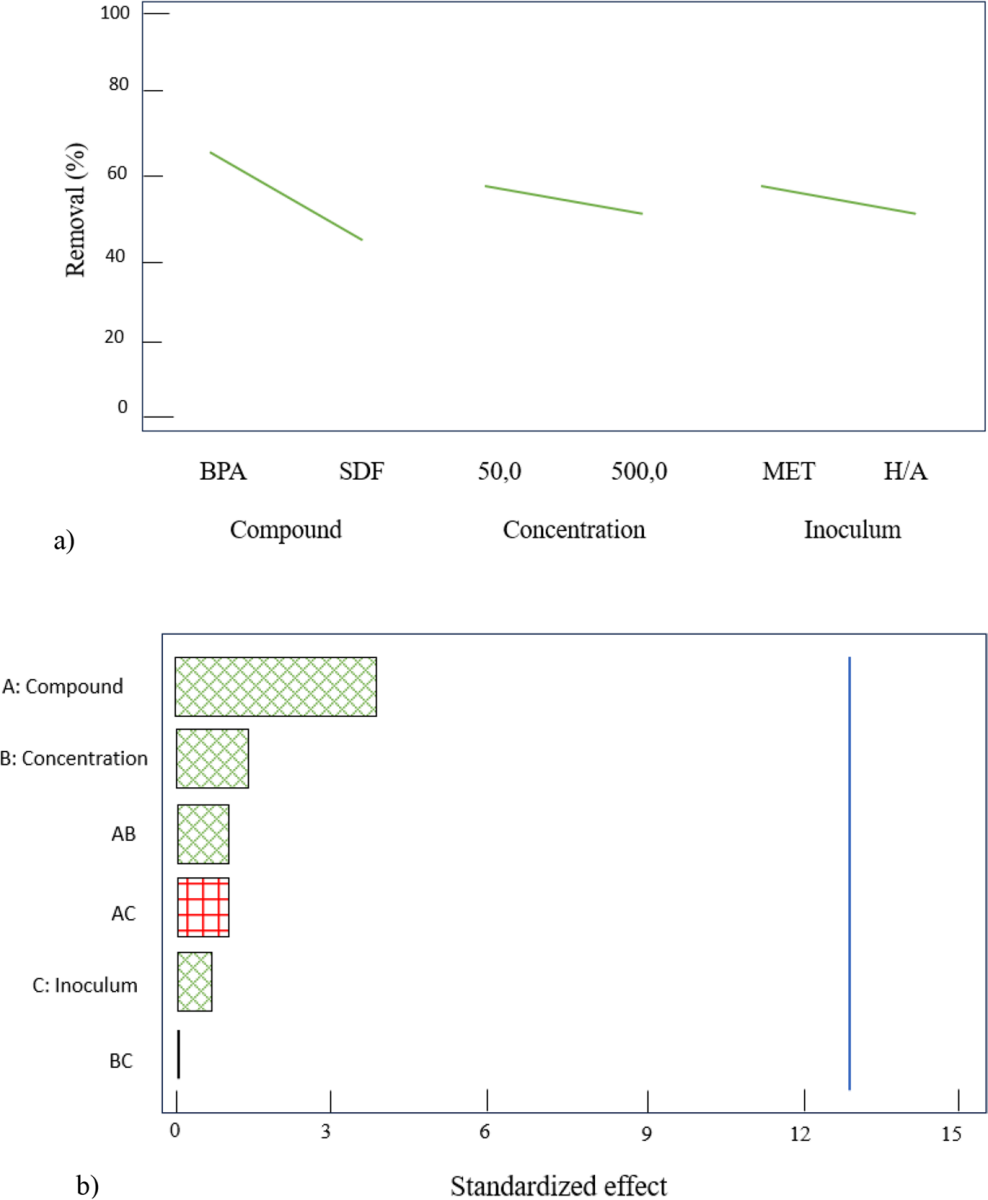
**a** Main effects plot. **b** Pareto chart for removal (%) using an anaerobic batch process. Compound = BPA, SDF; concentration = 50, 500 μg L^−1^; inoculum = H/A, MET. Green: negative effect, red: positive effect

Figure 5 summarizes the results obtained for the removal of BPA and SDF in sample tests treated with H/A and MET inoculum, respectively, after 28 days. The observed behavior suggests that an increase in BPA concentration had no effect on the removal obtained; the same behavior was identified in results with methanogenic inoculum. Furthermore, tests with hydrolytic inoculum showed a similar pattern, except of experiments with an initial concentration of 50 μg/L BPA (BPA_50_H/A), where the average removal was lower than that obtained in the other tests with the same compound. This fact was related to the statistical dispersion of the data in these experimental units and its significance was later evaluated by a multiple range test (Figure S5). Moreover, the average values obtained for the removal of SDF with MET inoculum suggests that an increase of sildenafil concentration had no effect on the removal obtained, like the behavior observed with BPA. Nevertheless, experiments with H/A inoculum and an initial concentration of 50 μg/L SDF (SDF_50_H/A) showed an average removal higher than that obtained in the other trials with the same compound; its significance was later evaluated by a multiple range test (Figure S6). In addition, Fig. 5 shows that average removal obtained for SDF was lower in most trials than those calculated in BPA tests, which suggests that this type of compound is a factor that could influence the removal, the significance of which was evaluated by the main effects diagram and a Pareto chart (Fig. 6).

Multiple range tests were used to evaluate the significance between mean averages obtained; for this a Tukey’s pairwise test was carried out. The results obtained indicate that there were no statistically significant differences between the pairs of means, with a 95.0% confidence level. Figure S5 and Figure S6 (see Supplementary Information) show the graphic results of the test performed. This means that concentrations in the range evaluated of 50–500 μg/L had no differential effect on the removal of BPA and SDF. Additionally, the results suggest that under the experimental conditions the type of inoculum had no significant effect on the removal of PPCPs with a 95.0% confidence level.

The effect of the factors evaluated in factorial design is shown in the main effects plot and the Pareto chart (Fig. 6). It is important to note that the Pareto chart indicates the magnitude and significance of the effect of each evaluated factor, as well as the interaction of these in the response variable (Rubio-Clemente et al., 2019), in this case, the removal percentage. In the Pareto chart, the reference line (vertical line) indicates the critical value, so any effect that exceeds this line can be considered significant. The positive/negative signs indicate whether the main and interaction effects influence the response positively or negatively, respectively. According to the results, the factors evaluated showed a negative effect on the response variable, except for the interaction of the factor “AC.” However, none of the factors had a significant effect on the percentage of removal (95.0% confidence level), which is consistent with the results obtained in multiple range tests.

### Mass balance

Figure S8 and Figure S9 show the overall transformation of COD at the end of experiments, where COD_biodegradable_ indicates the fraction of COD partially or completely transformed by biological mechanisms, and COD_recalcitrant_ indicates the fraction of COD that could not be degraded by microorganisms. A mass balance was performed according to the COD balance diagram for anaerobic digestion processes (Figure S7). The variables measured in the experiment were transformed to COD terms using conversion factors to carry out the balance.

The results of the mass balance for H/A inoculum experiments indicate that part of the substrate transformation occurred through biological mechanisms. Figure S8 shows that substrate biodegradation was partial in samples M1, M2, M3, and M4. Substrate transformation was confirmed by quantification of VFAs, intermediates of anaerobic digestion. The presence of COD_VFA_ in the mass balance for control and sample tests is an indicator of metabolism for acidogenic microorganisms. Additionally, according to the mass balance, there was recalcitrant COD at the end of the experiment. It is important to note that the main substrate used in this study was biodegradable, so this could be attributed to the methodological difference in the quantification of variables that are part of the mass balance. This aspect has been previously reported in COD balances for anaerobic digestion processes (Rodriguez, 2015).

Mass balance results for MET inoculum experiments (Figure S9) show that only part of the substrate was transformed by biological mechanisms. Additionally, the concentration of COD_VFA_ and COD_biodegradable_ in control and sample tests at the end of the experiment suggests that accumulation of VFAs occurred in these experimental units. This probably generated an imbalance in microorganisms present in the medium and the subsequent inhibition of methanogenic microorganisms at some time during the experiment. This was reflected in the incomplete transformation of VFAs to methane. On the other side, recalcitrant COD quantified in these experiments was attributed to the same factors mentioned in the experiments with H/A inoculum.

## Conclusions

The removal percentages obtained with BPA for H/A and MET biomass treatments were mainly related to sorption and abiotic mechanisms, such as hydrolysis. In contrast, biodegradation was the predominant mechanism for SDF removal under the experimental conditions of this study. Nevertheless, the degradation was partial, with percentages lower than 43% in tests with hydrolytic/acidogenic inoculum and lower than 41% in the tests with methanogenic inoculum.

On the other hand, statistical tests indicated that the factors evaluated had no significant effect on BPA and SDF removal in the evaluated range, with a 95.0% confidence level. In the case of SDF, which the removal obtained was attributed to a biodegradation process**,** the results suggested that hydrolysis/acidogenesis phase is a main contributor to biodegradation.

Finally, the results indicate that contact of biomass with each micropollutant did not generate adverse (toxic or inhibitory) effects on the microbial populations in the concentration range of 50–500 μg/L. Hydrolytic/acidogenic biomass inhibition was verified with acidogenic control tests, where the concentration of produced VFAs was similar to the concentration produced in sample tests with BPA and SDF. Moreover, methanogenic biomass inhibition was evaluated with control tests, particularly with TOC removal, where substrate removal was similar in both control and samples, with BPA and SDF. However, low substrate removal (highly biodegradable) indicates that there was probably inhibition of methanogenic microorganisms for some of the time of the experiment in control and sample tests, probably due to the accumulation of VFAs in the medium.

### Supplementary information

ESM 1 (DOCX 189 kb)

## Data Availability

The datasets generated during and analyzed during the current study are available from the authors on reasonable request.
