# Supplementary material for: Role of the hydrolytic-acidogenic phase on the removal of bisphenol A and sildenafil during anaerobic treatment
Source: Environ Monit Assess. 2023 Nov 30;195(12):1552. doi: 10.1007/s10661-023-12009-8 (PMC10689534; doi:10.1007/s10661-023-12009-8)
Supplement: Supplementary file 1 — (DOCX 189 kb) [file 10661_2023_12009_MOESM1_ESM.docx]

**Role of the hydrolytic-acidogenic phase on the removal of bisphenol A and sildenafil during anaerobic treatment**

**Supplementary Material**

J. Arcila-Saenz^1*^, G. Hincapié-Mejía^2^, Y. A. Londoño ^1^, G. A. Peñuela^1^

^1^GDCON Research Group, Faculty of Engineering, University Research Headquarters (SIU), University of Antioquia, Street 70 # 52-21, Medellín, Colombia

^2^ Environment, Habitat and Sustainability Research Group, University Institution Colegio Mayor de Antioquia, Street 78 # 65 – 46. Medellín, Colombia

* Corresponding author: Jennifer Arcila-Saenz. E-mail address: Jennifer.arcila@udea.edu.co

- **pH analysis**

Figure S1. Behavior of pH in control and samples with H/A inoculum after 28 days


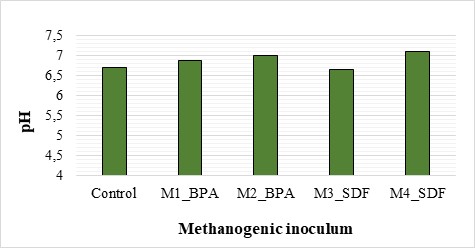


Figure S2. Behavior of pH in control and samples with MET inoculum after 28 days

- **Volatile fatty acids analysis**


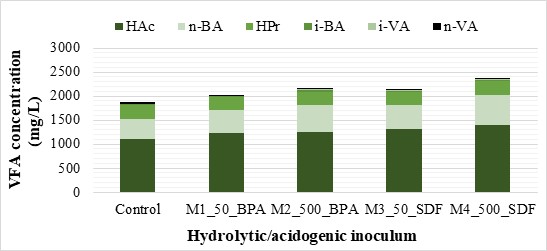


Figure S3. VFA concentration in control and samples with H/A inoculum after 28 days


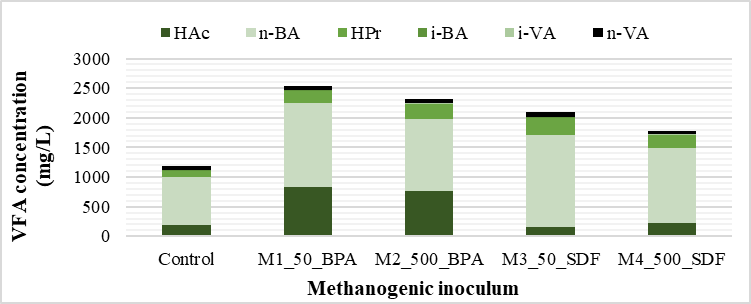


Figure S4. VFA concentration in control and samples with MET inoculum after 28 days

### **Effect of factors on PPCPs removal**


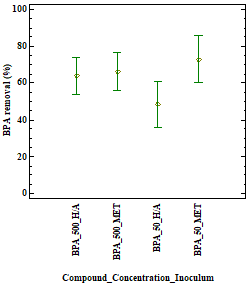


Figure S5. Multiple range test plot for BPA experiments.


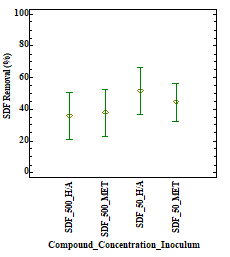


Figure S6. Multiple range test plot for SDF experiments.

## Mass balance


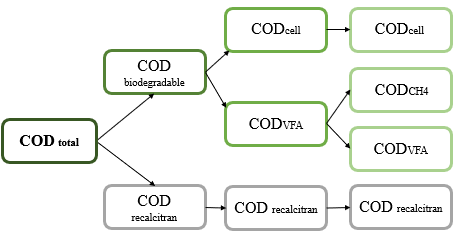


**Abbreviations:**

**Dissolved organic carbon (COD)**

**COD_cel_:** COD fraction of the influent converted into new cells. **COD_VFA_:** COD fraction present as VFAs in the effluent. **COD_CH4_:** COD fraction of the influent converted to methane. **COD_rec_:** COD fraction which can not be degraded by microorganisms.

Figure S7. Diagram of the COD balance in anaerobic degradation processes. Adapted from (Rodriguez 2015)


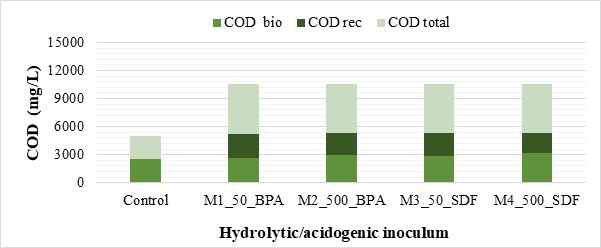


Figure S8. COD balance for experiments with H/A inoculum


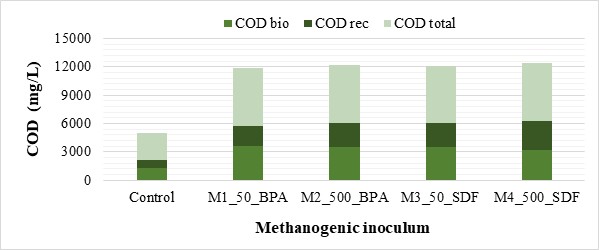


Figure S9. COD balance for experiments with MET inoculum
